# Mixed short-chain fatty acids and high-fiber diet reshape SCFA profiles and attenuate post-infarction cardiac fibrosis in mice

**DOI:** 10.3389/fcvm.2026.1858770

**Published:** 2026-07-23

**Authors:** Zhiyuan Xu, Qi Zhong, Shunrong Zhao, Kaidi Zhao, Junyi Zhang

**Affiliations:** 1 Department of Cardiology, Yantai Yuhuangding Hospital, Qingdao University, Yantai, China; 2 Department of Cardiology, Qingdao Hospital, University of Health and Rehabilitation Sciences (Qingdao Municipal Hospital), Qingdao, China

**Keywords:** BNP, cardiac fibrosis, cTnT, high-fiber diet, myocardial infarction, short-chain fatty acids, targeted metabolomics

## Abstract

**Background:**

Short-chain fatty acids (SCFAs) are microbiota-derived metabolites implicated in immunometabolic regulation. How their systemic and myocardial signatures diverge during post-infarction remodeling, and how these signatures relate to cardiac fibrosis, remains poorly characterized.

**Methods:**

Permanent LAD ligation was performed in male C57BL/6J mice assigned to five groups: Control (G0), Sham (G1), MI (G2), MI with mixed SCFA gavage from postoperative day 1 (G3), and MI with a high-fiber diet (HFF, G4). Serum and heart tissue were harvested at day 7 and day 28 (*n* = 5 per group per time point). Targeted SCFA profiling was performed in both compartments. Cardiac fibrosis was evaluated by Masson staining, immunohistochemistry, and qPCR; myocardial energy status by ATP content and NAD⁺/NADH ratio; and circulating biomarkers by ELISA. Pathway-related transcripts were measured by qPCR.

**Results:**

Acetic acid predominated across all groups. MI was associated with an altered serum SCFA profile, and serum profiles separated more clearly by PCA than myocardial profiles, in which hierarchical clustering highlighted a prominent HFF-related shift. MI increased cardiac collagen deposition, an effect attenuated by both SCFA supplementation and HFF, with Collagen III markedly elevated after MI and reduced in the intervention groups. MI caused pronounced ATP depletion at both time points, partially restored by both interventions and more so by HFF. The NAD⁺/NADH ratio was more variable at day 7 and converged across groups by day 28. Serum cTnT and BNP rose after MI and were lower in the intervention groups, whereas serum TGF-*β*1 did not differ significantly.

**Conclusions:**

Increasing SCFA availability through mixed SCFA supplementation or a high-fiber diet was associated with divergent serum-vs.-cardiac SCFA remodeling, reduced post-MI fibrosis, and improved myocardial energy balance.

## Highlights

- Targeted profiling reveals distinct serum vs. myocardial SCFA patterns during post-MI remodeling.
- Mixed SCFAs gavage and high-fiber diet attenuate cardiac fibrosis, with reduced Collagen III after MI.
- Both interventions restore myocardial ATP; HFF produces more pronounced recovery.
- MI-associated elevations in serum cTnT and BNP are lower in the intervention groups.

## Background

Myocardial infarction (MI) remains a leading cause of heart failure and long-term cardiovascular morbidity. Clinical outcomes depend heavily on the magnitude and persistence of post-infarction left ventricular remodeling (1). Cardiac fibrosis occupies a central position in this process: early fibroblast activation and extracellular matrix (ECM) deposition stabilize the infarct scar, but sustained activation becomes maladaptive, contributing to ventricular stiffening, arrhythmogenesis, and progressive functional decline (1, 2). At present, no approved therapies directly target ECM accumulation or fibroblast activation in routine clinical practice (1), and the time-dependent, multicellular regulation of post-MI fibrosis continues to pose challenges for therapeutic development (2).

MI also imposes severe metabolic stress on the surviving myocardium. Energy supply-demand mismatch and impaired substrate utilization amplify cellular injury and influence repair trajectories. AMP-activated protein kinase (AMPK) integrates metabolic and oxidative stress signals across ischemia, hypertrophy, and heart failure, though its regulation is isoform-, stage-, and context-dependent (3). Short-chain fatty acids (SCFAs) have drawn attention as alternative cardiac substrates because they bypass CPT1-dependent mitochondrial transport and can be efficiently oxidized in the stressed heart, contributing meaningfully to oxidative ATP generation (4). Whether augmenting SCFA availability can improve myocardial energetics and in turn attenuate fibrotic remodeling is therefore a question of both mechanistic and practical significance.

SCFAs are central mediators of the gut–heart axis, in which diet, microbiota composition, epithelial barrier function, and microbial metabolites collectively modulate systemic inflammation and host metabolism in cardiovascular disease (5–9). Acetate, propionate, and butyrate—generated by bacterial fermentation of dietary fiber—act on host physiology through G-protein–coupled receptors, histone deacetylase inhibition, and acetyltransferase-mediated acetylation programs (10–13). Disrupted circulating SCFA profiles have been documented in heart failure patients, underscoring the translational relevance of microbiota–metabolite dysregulation to cardiac disease (7, 14). In experimental MI models, microbiome configurations enriched for butyrate-producing bacteria improve post-infarction responses, and engineered probiotics designed to sustain circulating SCFA levels reduce myocardial injury in ischemia–reperfusion settings through immunomodulatory mechanisms (15, 16). High-fiber feeding likewise attenuates post-MI inflammation and adverse remodeling by reshaping gut microbial ecology and metabolite production (17), and individual SCFAs—propionate and butyrate—reduce post-infarction dysfunction and myocardial fibrosis through mechanisms linked to macrophage polarization and mitochondrial function (18, 19).

Two questions directly relevant to mechanism and translation remain open despite this accumulated evidence. First, although prior studies have characterized fecal or circulating SCFA changes after MI, the relationship between serum SCFA shifts and myocardial SCFA signatures has not been systematically examined, despite the likelihood of differential regulation between these compartments (5, 7–9). Second, whether SCFA-directed interventions simultaneously improve myocardial energy indices—particularly ATP availability—and modulate the profibrotic signaling networks that govern fibroblast activation and ECM accumulation has not been tested (1–3). To address these gaps, we performed targeted SCFA profiling in paired serum and cardiac tissue from mice subjected to permanent LAD ligation and compared two clinically relevant strategies—oral mixed SCFA supplementation and high-fiber feeding—with assessments at early (day 7) and chronic remodeling (day 28) time points.

## Methods

### Animals and ethics

Male C57BL/6J mice (20–25 g) were housed under specific pathogen-free conditions (12-h light/dark cycle; controlled temperature and humidity) with free access to food and water. All animal procedures were approved by the Institutional Animal Care and Use Committee and conducted in accordance with relevant guidelines. Animals were randomly assigned to groups. Tissue processing and image quantification were performed blinded to group allocation whenever feasible.

Male C57BL/6J mice were purchased from Shandong Jinan Pengyue Laboratory Animal Breeding Co., Ltd. (Jinan, Shandong, China).

Animals were euthanized by cervical dislocation under deep isoflurane anesthesia. Mice were placed in an induction chamber with isoflurane 3%–4% in oxygen at a flow rate of 0.5–1 L/min. After loss of righting reflex, anesthesia was maintained with isoflurane 1.5%–2.5% via nose cone. The absence of pedal withdrawal reflex was confirmed prior to cervical dislocation, which was performed by trained personnel by quickly and firmly pulling the tail while holding the head to separate the cervical vertebrae and spinal cord, ensuring immediate death.

### Experimental design and grouping

Mice were allocated to five groups: G0 Control, G1 Sham, G2 MI, G3 MI + mixed SCFAs gavage, and G4 MI + high-fiber diet (HFF). MI or sham surgery was performed on day 0. Mixed SCFAs were administered by oral gavage starting on postoperative day 1 (D1) and continued daily until euthanasia. Animals were sacrificed at day 7 (D7) or day 28 (D28). Unless otherwise specified, *n* = 5 animals per group per time point were used for downstream analyses.

### LAD ligation model of myocardial infarction and sham surgery

MI was induced by permanent ligation of the left anterior descending (LAD) coronary artery. Mice were anesthetized with isoflurane, intubated, and mechanically ventilated. After left thoracotomy at the fourth intercostal space, the LAD was ligated with a 7–0 suture approximately 1–2 mm below the left atrial appendage. Successful ligation was confirmed by immediate blanching of the anterior left ventricular wall. In sham-operated animals, the suture was passed under the LAD but not tied. The chest was closed in layers and animals recovered on a warming pad. Postoperative monitoring and analgesia were provided according to institutional protocols.

### High-fiber diet intervention

Mice in the HFF group (G4) received a customized high-fiber chow beginning prior to surgery and continuing through the experimental period until euthanasia. The customized diet was supplemented with >5% inulin as a fermentable dietary fiber component. The diet was manufactured by Jiangsu Xietong Pharmaceutical Bioengineering Co., Ltd. All other groups received standard chow.

### Mixed SCFAs gavage

Mice in the MI + mixed SCFAs group (G3) received a mixed SCFA solution by oral gavage starting on D1 and continuing daily until sacrifice. Mice in the MI + mixed SCFAs group (G3) received a mixed SCFA solution by oral gavage starting on D1 and continuing daily until sacrifice. The mixed SCFA solution consisted of sodium propionate and sodium butyrate at equal molar concentrations. Specifically, sodium propionate and sodium butyrate were each prepared at 25 mM, resulting in a total SCFA concentration of 50 mM. A volume of 200 μL was administered to each mouse once daily, corresponding to 5 μmol sodium propionate and 5 μmol sodium butyrate per mouse per day. Control, sham, MI, and HFF groups received an equal volume of vehicle by gavage.

### Sample collection

Mice were euthanized at D7 or D28. Blood was collected at sacrifice and centrifuged to obtain serum, which was stored at −80 °C. Hearts were rapidly excised, rinsed in cold PBS, and processed for (i) paraformaldehyde fixation and paraffin embedding (histology/IHC), and (ii) snap-frozen storage for RNA extraction and biochemical assays. When applicable, intestinal samples were collected for histology and RNA extraction.

### Histology (H&E and masson trichrome)

Paraffin-embedded hearts were sectioned (4–5 μm). Hematoxylin and eosin (H&E) staining was performed for general morphology. Masson trichrome staining was used to evaluate collagen deposition. Fibrosis was assessed on stained sections using standardized acquisition settings.

### Immunohistochemistry (IHC) and quantification

Paraffin sections were deparaffinized, rehydrated, and subjected to antigen retrieval. Sections were incubated with primary antibodies against Collagen I, Collagen III, and *α*-SMA, followed by HRP-conjugated secondary antibodies and DAB development, with hematoxylin counterstaining. Quantification was performed as positive area ratio using ImageJ/Fiji under consistent thresholding rules; multiple fields were analyzed per section and averaged per animal.

### RNA extraction and quantitative real-time PCR

Total RNA from heart tissue was extracted using TRIzol-based methods and reverse-transcribed to cDNA. Quantitative PCR was performed with SYBR Green chemistry. Targets assessed in cardiac tissue included: TGF-*β*1, Smad2, Smad3, IL-6, Col1a1, Col3a1, and AMPK. Expression was normalized to an internal control gene (e.g., GAPDH), and relative expression was calculated using the 2^−*ΔΔ*Ct^ method. Technical replicates were performed for each biological sample.

### ELISA assays

Commercial ELISA kits were used to measure serum TGF-*β*1, serum cardiac troponin T (cTnT), and BNP. BNP was measured in both heart tissue homogenate and serum as indicated. Assays were performed according to the manufacturers’ instructions using appropriate standards and sample dilutions.

### Myocardial ATP and NAD⁺/NADH measurements

Myocardial ATP content was measured using a commercial assay kit and normalized to wet tissue weight (*μ*mol/kg wet tissue). The NAD⁺/NADH ratio was determined using an enzymatic cycling-based kit following the manufacturer's protocol. All measurements were performed with appropriate blanks and internal controls.

### Targeted metabolomics of SCFAs

Targeted SCFA profiling was performed in serum and heart tissue using a targeted metabolomics platform with internal standards. Data were processed according to platform procedures and used for multivariate analyses. PCA score plots and hierarchical clustering heatmaps were generated to visualize group-level patterns. Random forest analysis was applied to estimate feature importance for group discrimination based on the targeted SCFA panel.

### Supplementary immunoblot material

Full-length uncropped immunoblot images from exploratory assays performed during the study are provided as Additional file 1 for transparency. Because corresponding total AMPK and total Smad2/3 protein levels were not assessed, these immunoblots were not used for quantitative pathway analysis or to support mechanistic conclusions in the revised manuscript.

### Statistical analysis

Data are presented as mean ± SD unless stated otherwise. For comparisons among multiple groups, one-way ANOVA with appropriate multiple-comparison correction was used when test assumptions were met; otherwise, nonparametric tests were applied. A two-sided *P* < 0.05 was considered statistically significant. Analyses were performed using GraphPad Prism and/or R.

## Results

### 1. Study design and targeted SCFA profiling in serum and myocardium

**Figure 1:**
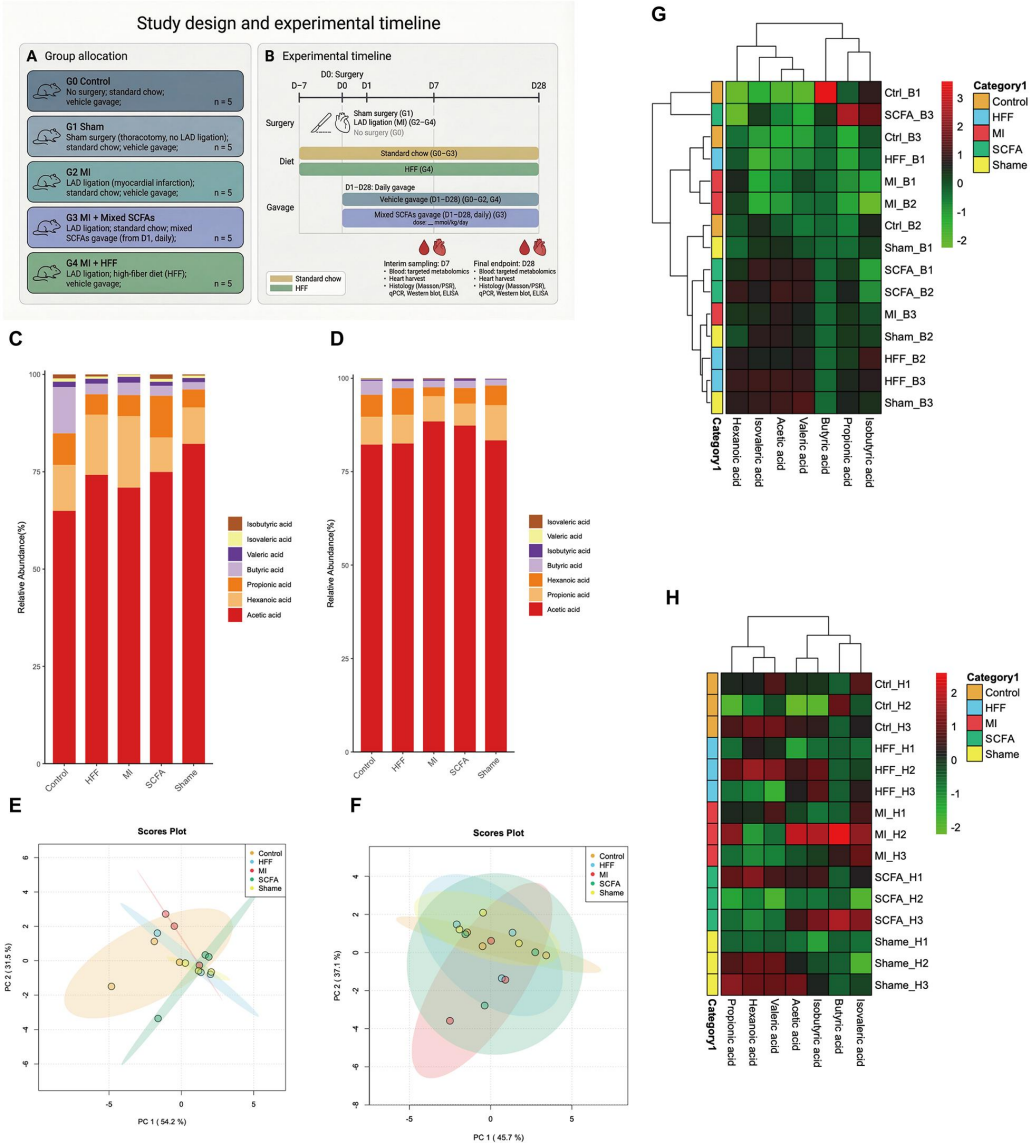
Study design and targeted SCFA profiling in serum and heart tissue. **(A)** Group allocation: Control (G0), Sham (G1), MI (G2), MI + mixed SCFAs gavage (G3), and MI + high-fiber diet (HFF; G4). *n* = 5 per group per time point. **(B)** Experimental timeline. MI or sham surgery was performed on day 0; mixed SCFAs were administered by oral gavage from day 1 to day 28. Samples were collected at day 7 (interim) and day 28 (final). **(C,D)** Stacked bar plots showing the relative abundance of individual SCFAs in serum **(C)** and heart tissue **(D) (E,F)** PCA score plots based on the targeted SCFA panel for serum **(E)** and heart tissue **(F) (G,H)** Unsupervised hierarchical clustering heatmaps of SCFA profiles in serum **(G)** and heart tissue **(H)** Panels C–H were used for descriptive and exploratory visualization; no inferential statistical test was applied to these panels.

Five groups were studied: Control (G0), Sham (G1), MI (G2), MI + mixed SCFAs gavage from postoperative day 1 (G3), and MI + high-fiber diet (HFF; G4), with blood and tissues collected at day 7 and day 28 (Figures 1A,B). Across both serum and cardiac tissue, acetic acid was the predominant SCFA in all groups, while relative proportions of minor SCFAs varied among conditions (Figures 1C,D). PCA based on the targeted SCFA panel showed more discernible group separation in serum than in cardiac tissue, where substantial overlap persisted across groups (Figures 1E,F). Hierarchical clustering further indicated an MI-associated shift in the serum SCFA pattern, whereas the myocardial heatmap showed distinct clustering in the HFF group relative to other conditions, consistent with differential SCFA handling between the two compartments (Figures 1G,H).

### 2. Mixed SCFAs and HFF attenuated post-MI cardiac fibrosis

**Figure 2:**
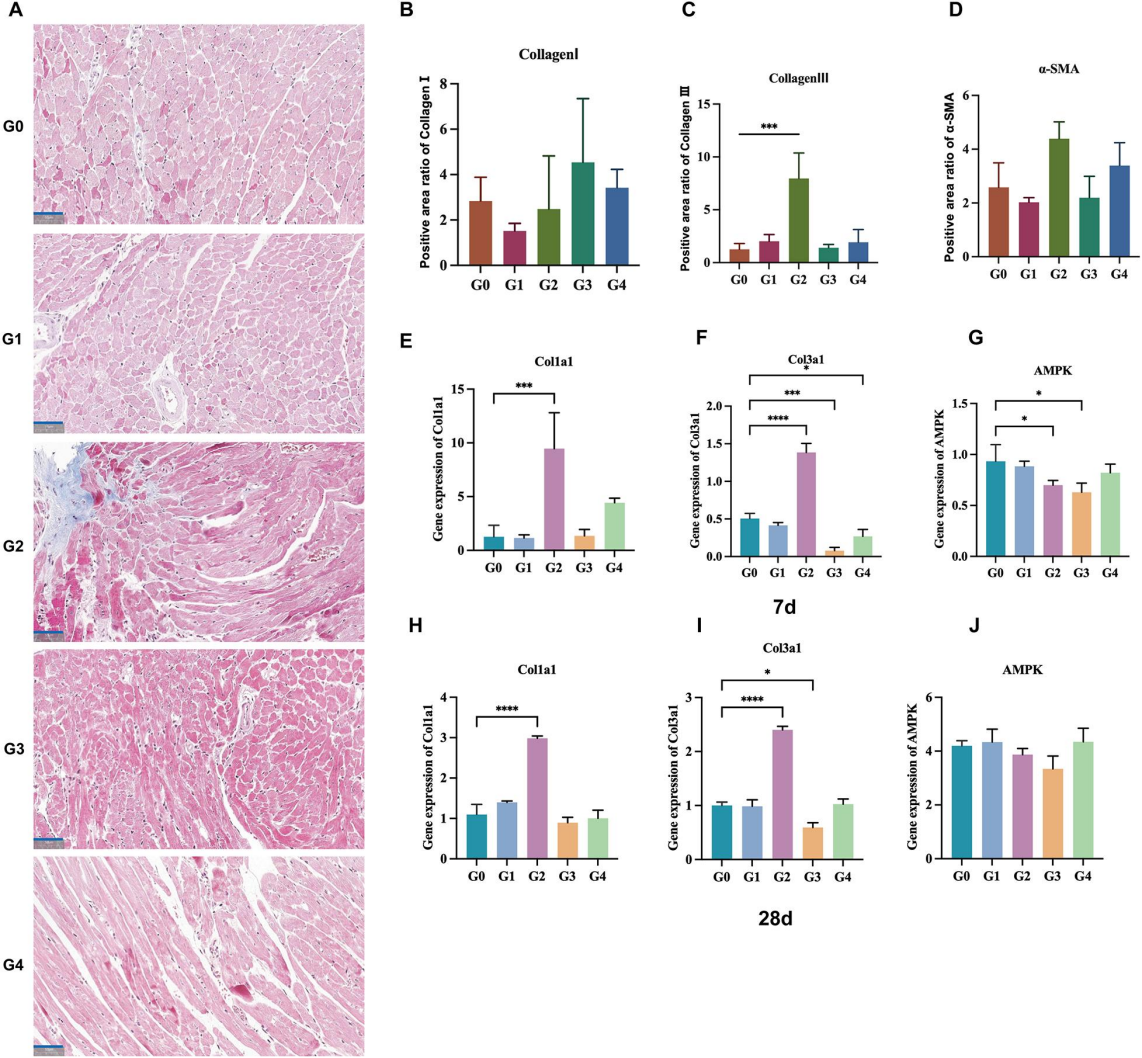
Mixed SCFAs and HFF attenuate post-MI cardiac fibrosis. **(A)** Representative Masson trichrome staining of cardiac sections at day 28 (collagen in blue) across groups (G0–G4). **(B–D)** Quantification of immunohistochemistry positive area ratios for Collagen I **(B)**, Collagen III **(C)**, and *α*-SMA **(D) (E–G)** Cardiac mRNA expression of Col1a1 **(E)**, Col3a1 **(F)**, and AMPK **(G)** at day 7. **(H–J)** Cardiac mRNA expression of Col1a1 **(H)**, Col3a1 **(I)**, and AMPK **(J)** at day 28. Data are shown as mean ± SD; error bars represent SD. Unless otherwise indicated, *n* = 5 biological replicates per group per time point. Statistical comparisons among groups were performed using one-way ANOVA followed by Tukey's multiple-comparisons test. Statistical significance is indicated in the panels.

Masson trichrome staining demonstrated increased collagen deposition in MI hearts (G2) compared with controls; both interventions attenuated fibrotic features in representative sections, with HFF (G4) appearing closer to controls than MI (Figure 2A). IHC quantification showed that Collagen III was markedly increased in MI and lower in the intervention groups (Figure 2C). By contrast, Collagen I did not show a consistent MI-associated increase and exhibited substantial variability across groups (Figure 2B). *α*-SMA tended to be higher in MI than in controls, while intervention groups showed partial attenuation without clear statistical separation (Figure 2D).

At the transcript level, MI induced a robust increase in Col1a1 at day 7, which was reduced in the SCFA (G3) and HFF (G4) groups (Figure 2E). Col3a1 followed a similar pattern of MI-associated elevation at day 7 with lower expression in intervention groups (Figure 2F). AMPK mRNA showed modest group differences at day 7 (Figure 2G). At day 28, Col1a1 and Col3a1 remained elevated in MI relative to controls and were lower in both intervention groups (Figures 2H,I), whereas AMPK transcript levels were broadly comparable among groups at this time point (Figure 2J).

### 3. Interventions improved myocardial energy status after MI

**Figure 3:**
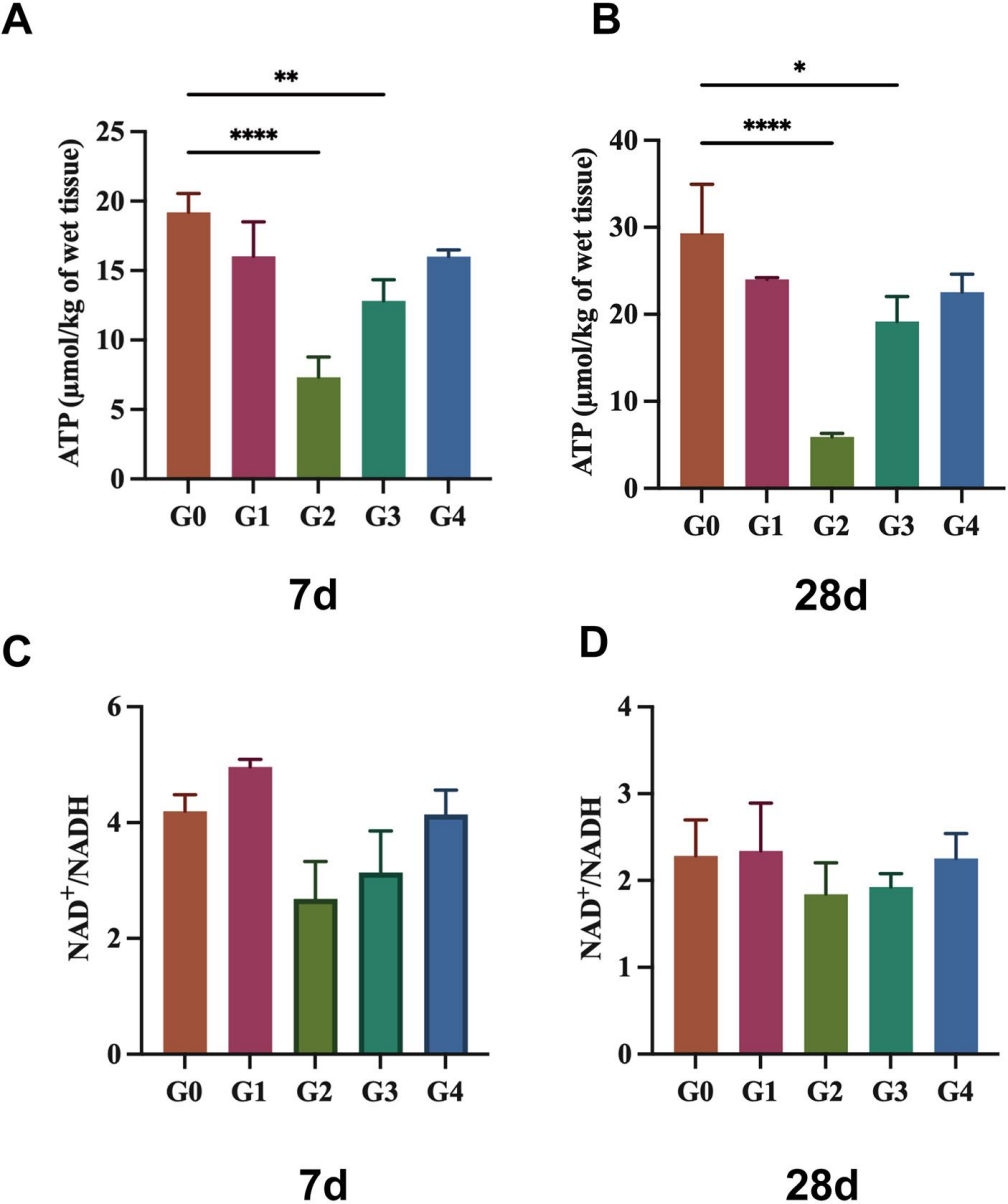
Interventions improve myocardial energy status after MI. **(A,B)** Myocardial ATP content at day 7 **(A)** and day 28 **(B) (C,D)** Myocardial NAD⁺/NADH ratio at day 7 **(C)** and day 28 **(D)** Data are shown as mean ± SD; error bars represent SD. *n* = 5 biological replicates per group per time point. Statistical comparisons among groups were performed using one-way ANOVA followed by Tukey's multiple-comparisons test. Statistical significance is indicated in the panels.

MI substantially reduced myocardial ATP content at both day 7 and day 28 (Figures 3A,B). Both interventions raised ATP relative to MI, with HFF (G4) showing more pronounced restoration toward control levels across time points (Figures 3A,B). The NAD⁺/NADH ratio showed a lower mean in MI at day 7 with variability across groups, and values converged among groups at day 28 (Figures 3C,D), indicating that ATP was the more reliable energetic readout in this dataset.

### 4. Circulating biomarkers, cardiac transcriptional changes, and SCFA feature importance

**Figure 4:**
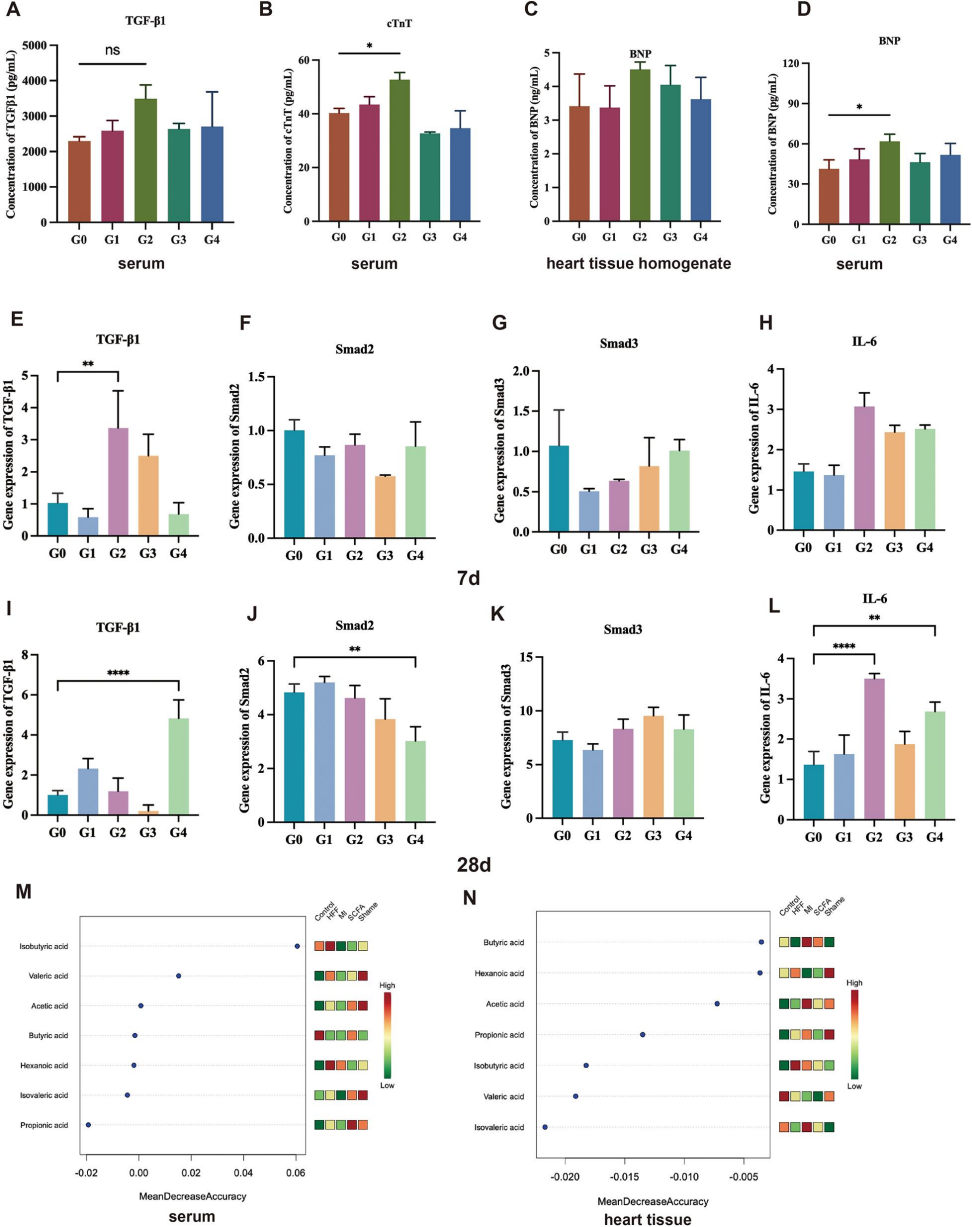
Circulating biomarkers, cardiac transcriptional changes, and SCFA feature importance after MI. **(A–D)** ELISA measurements of TGF-*β*1 (serum; A), cTnT (serum; **B**), BNP (heart tissue homogenate; **C**), and BNP (serum; **D**). **(E–H)** Cardiac mRNA expression of TGF-*β*1 **(E)**, Smad2 **(F)**, Smad3 **(G)**, and IL-6 **(H)** at day 7. **(I–L)** Cardiac mRNA expression of TGF-*β*1 **(I)**, Smad2 **(J)**, Smad3 **(K)**, and IL-6 **(L)** at day 28. **(M,N)** Random forest feature-importance analysis using Mean Decrease Accuracy for targeted SCFAs in serum **(M)** and heart tissue **(N)** Data are shown as mean ± SD; error bars represent SD. Unless otherwise indicated, *n* = 5 biological replicates per group per time point. For quantitative comparisons in panels **A–L**, statistical comparisons among groups were performed using one-way ANOVA followed by Tukey's multiple-comparisons test. Panels **M,N** show random forest feature-importance rankings and were not subjected to inferential statistical testing. Statistical significance is indicated in the panels.

By ELISA, serum TGF-*β*1 did not differ significantly across groups (Figure 4A). Serum cTnT was elevated in MI relative to controls and lower in the intervention groups (Figure 4B). BNP in heart-tissue homogenate varied across groups but did not reach significance (Figure 4C), whereas serum BNP was increased in MI and lower in the intervention groups (Figure 4D).

At the transcript level, TGF-*β*1 mRNA was increased in MI at day 7 and lower in the HFF group (Figure 4E). Smad2 and Smad3 transcripts varied modestly across groups at day 7 (Figures 4F,G), while IL-6 was increased in MI (Figure 4H). At day 28, TGF-*β*1 transcripts were instead higher in the HFF group (Figure 4I), whereas Smad2 was lower in HFF than in control/sham (Figure 4J). Smad3 varied modestly across groups at day 28 (Figure 4K). IL-6 remained highest in MI at day 28 and was lower in the intervention groups (Figure 4L). Random forest feature-importance analysis of the serum SCFA panel ranked isobutyric acid as the top metabolite for group discrimination (Figure 4M), whereas the myocardial model produced a different ranking (Figure 4N).

Jejunal histology and intestinal tight-junction gene expression data are provided in Supplementary Figure S3, S4. Unless otherwise stated, *n* = 5 animals per group per time point (day 7 and day 28).

## Discussion

We profiled SCFAs in paired serum and cardiac tissue and compared oral mixed SCFA supplementation with high-fiber feeding (HFF) at early (day 7) and chronic (day 28) stages of post-MI remodeling. Group separation was clearer for serum than for myocardial profiles, indicating that the circulating and cardiac SCFA compartments are not interchangeable. This compartmental distinction has rarely been examined in prior gut–heart axis studies (5, 7, 9). Both interventions attenuated fibrotic remodeling, as shown by Masson staining and by the significant reduction in Collagen III immunoreactivity after SCFA gavage. MI also produced a consistent myocardial ATP deficit that both interventions partially restored, with the stronger recovery seen under HFF.

The stronger ATP recovery in the HFF group may reflect the difference between continuous dietary modulation and bolus supplementation. Gavage delivers a defined dose of exogenous SCFAs, whereas dietary fiber drives sustained endogenous production through microbial fermentation and reshapes the wider gut metabolic environment. Beyond acetate, propionate, and butyrate, HFF may affect intestinal barrier integrity, systemic inflammatory tone, and other microbiota-derived metabolites not captured by our targeted panel. Together, these effects could produce a metabolic and inflammatory environment more favorable to the infarcted myocardium than short-term delivery of selected SCFAs. The greater ATP restoration under HFF should therefore not be read as evidence that fiber acts solely by raising SCFA levels; instead, fiber-driven remodeling of the gut–heart axis may support myocardial energy recovery through several converging routes. This is consistent with the established view of post-MI fibrosis as a time-dependent, multicellular process and an important therapeutic target for which no approved ECM-directed therapy yet exists (1, 2).

SCFAs can shape post-MI remodeling through both metabolic and signaling routes. As carbon substrates, they bypass CPT1-dependent mitochondrial transport and feed oxidative phosphorylation in the energy-deficient heart, providing a direct metabolic basis for the ATP recovery seen in both groups (4). As signaling molecules, they act through GPCRs, HDAC-related epigenetic regulation, and acetyltransferase-mediated acetylation (10–13), routes that together can reshape profibrotic gene expression. AMPK links energetic stress to tissue remodeling, and its regulation in cardiovascular disease depends on isoform, disease stage, and context (3); in our data AMPK transcripts varied with time, although pathway activation at the protein level was not assessed. TGF-*β*/Smad signaling is a canonical driver of fibroblast activation and ECM accumulation after MI (2), and TGF-*β*1, Smad2, and Smad3 transcripts likewise changed across groups and time points, consistent with involvement of this pathway in remodeling under SCFA supplementation or HFF. These transcript-level findings are best read as pathway-associated observations rather than evidence of causal suppression: their time- and group-dependent pattern fits dynamic regulation within the repair network better than a uniform change across all pathway components.

The compartment-specific SCFA pattern we observed carries a practical implication for the gut–heart axis literature. Several reviews point to gut epithelial permeability, systemic inflammation, and diet-derived microbial metabolites as drivers of heart failure progression, with SCFAs among the most plausible mediators (5–9). Clinically, circulating acetate and propionate are reduced across the heart failure spectrum, while butyrate changes vary by ejection fraction subtype (7, 14). In MI specifically, microbiomes enriched in butyrate-producing bacteria have been reported to improve post-infarction outcomes (15), and probiotics engineered to sustain plasma SCFA levels can reduce myocardial injury alongside changes in inflammatory and macrophage-related pathways (16). Dietary modulation has likewise been linked to reduced post-MI inflammation and adverse remodeling via gut microbial and metabolite changes (17), and individual SCFAs such as propionate and butyrate have been reported to improve post-infarction dysfunction and fibrosis in association with macrophage polarization and mitochondrial homeostasis. Because we did not profile immune cells, however, these reports should be taken as context rather than as direct evidence that macrophage polarization mediated our findings (18, 19). Our paired serum–cardiac data suggest that myocardial SCFA pools are buffered by local uptake, utilization, perfusion, and metabolic remodeling, which may dampen tissue-level PCA separation even when systemic shifts are pronounced. This bears on biomarker development and dosing strategy in future interventional work.

The study has several limitations. Within-group variability was substantial for some IHC and ELISA readouts, which reduced our power to detect differences in those endpoints and calls for cautious interpretation. Although AMPK- and TGF-*β*/Smad-related transcripts varied across groups and time points, our data do not establish that AMPK activation or TGF-*β*/Smad inhibition is required for the protective effects of SCFA supplementation or HFF (20, 25). Corresponding total AMPK and total Smad2/3 protein levels were not assessed; therefore, phosphorylated protein signals were not used for quantitative pathway interpretation or mechanistic conclusions in the revised manuscript. We also did not assess immune-cell composition, flow cytometry, or macrophage polarization, so any discussion of immunomodulatory mechanisms is inferential and rests on prior literature rather than on direct evidence here. Pharmacological inhibition or activation, genetic perturbation of AMPK or Smad signaling, properly normalized phospho/total protein analyses, and cell-type-resolved studies will be needed to establish causal involvement. We therefore regard these pathway-related findings as associative rather than definitive. Given the central role of macrophages in post-MI remodeling, future work using receptor perturbation, cell-resolved transcriptomics, or targeted immune profiling would further strengthen mechanistic inference (6, 21–24).

## Conclusions

Oral mixed SCFA supplementation and high-fiber feeding each reshaped systemic and myocardial SCFA profiles, reduced post-MI fibrotic burden, and partially restored myocardial ATP content. The divergence between serum and cardiac SCFA profiles shows that compartment-specific regulation cannot be inferred from systemic measurements alone. These findings strengthen the rationale for SCFA-centered dietary strategies after MI and point to energetic recovery and profibrotic remodeling as priorities for future mechanistic and functional studies, including validation of AMPK- and TGF-*β*/Smad-related candidate pathways.

## Data Availability

The original contributions presented in the study are included in the article/Supplementary Material, further inquiries can be directed to the corresponding author.

## Supplementary material

Supplementary Figure S1 Cardiac IHC images for fibrotic markers. Representative sections from G0–G4 stained for Collagen I (**A**), Collagen III (**B**), and α-SMA (**C**). Sections were counterstained with hematoxylin. Scale bars as indicated.

Supplementary Figure S2 Cardiac H&E staining. Representative H&E-stained sections illustrating myocardial morphology across groups (G0–G4). Scale bars as indicated.

Supplementary Figure S3 Jejunal histology. Representative H&E-stained jejunal sections from G0–G4. Scale bars as indicated.

Supplementary Figure S4 Intestinal tight-junction gene expression. qPCR quantification of Claudin-1 (**A, D**), Occludin (**B, E**), and ZO-1 (**C, F**) in jejunal tissue at day 7 (**A–C**) and day 28 (**D–F**). Data are shown as mean ± SD; n=5 biological replicates per group per time point. Statistical comparisons among groups were performed using one-way ANOVA followed by Tukey’s multiple-comparisons test. Statistical significance is indicated in the panels.

Supplementary Table S1 Antibodies used for immunohistochemistry and supplementary exploratory immunoblotting.

Supplementary Table S2 Primer sequences used for quantitative real-time PCR.
