# Supplementary material for: Mixed short-chain fatty acids and high-fiber diet reshape SCFA profiles and attenuate post-infarction cardiac fibrosis in mice
Source: Front Cardiovasc Med. 2026 Jul 23;13:1858770. doi: 10.3389/fcvm.2026.1858770 (PMC13441702; doi:10.3389/fcvm.2026.1858770)
Supplement: Supplementary file 1 [file Datasheet1.pdf]

Supplementary materials

western blotting

Figure 3E

Heart tissue collected 28 days post-MI (D28)

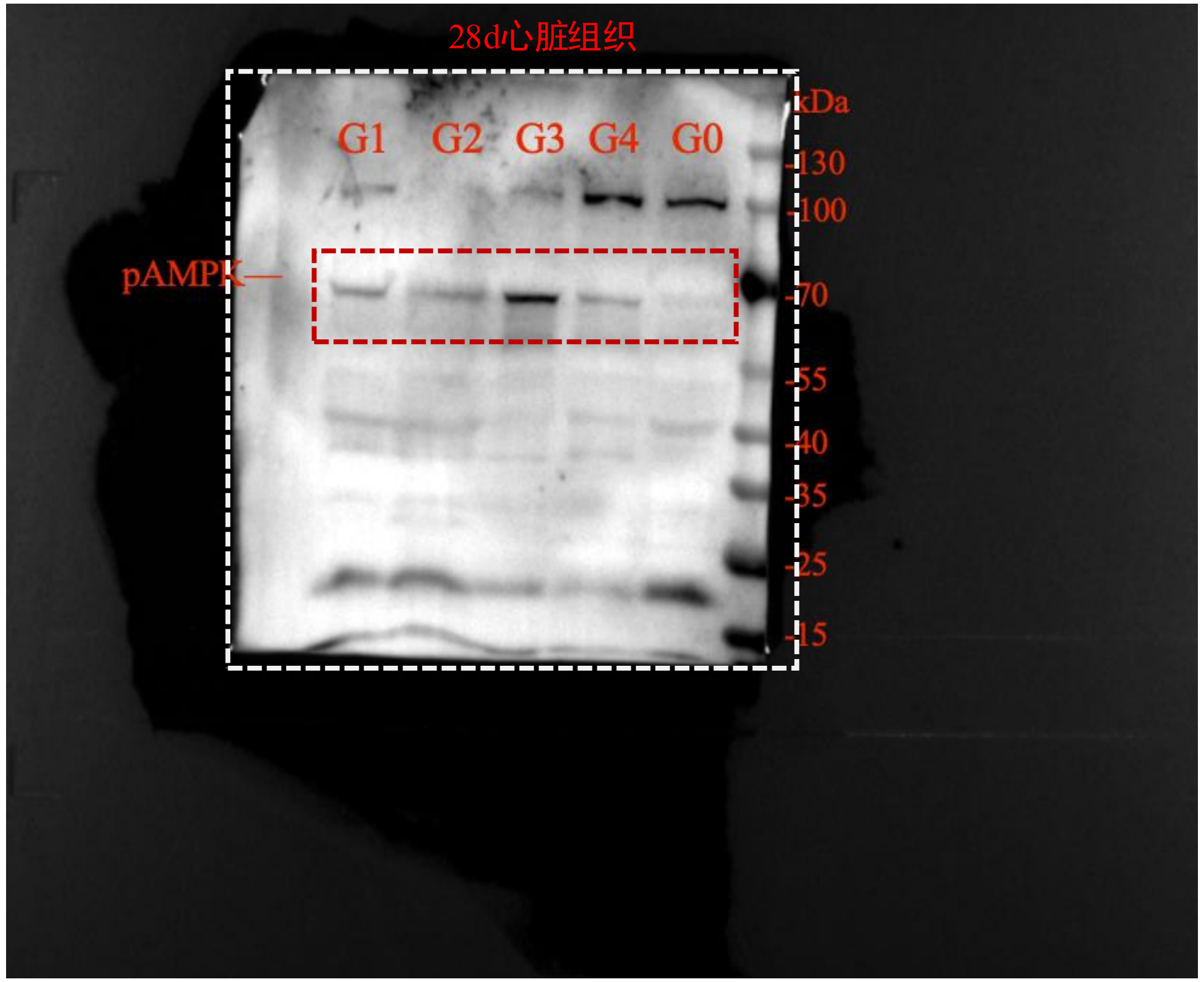

pAMPK

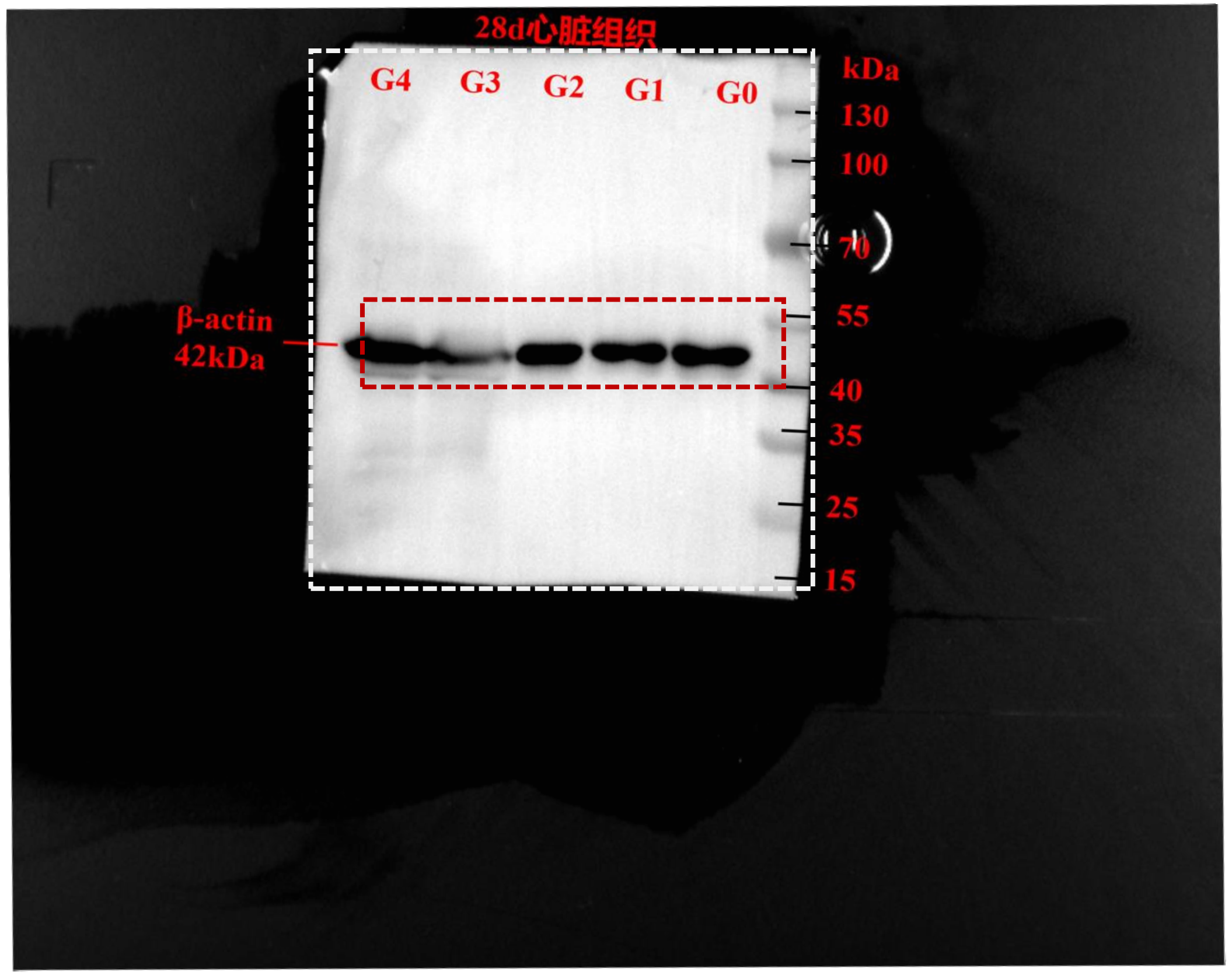

β-actin

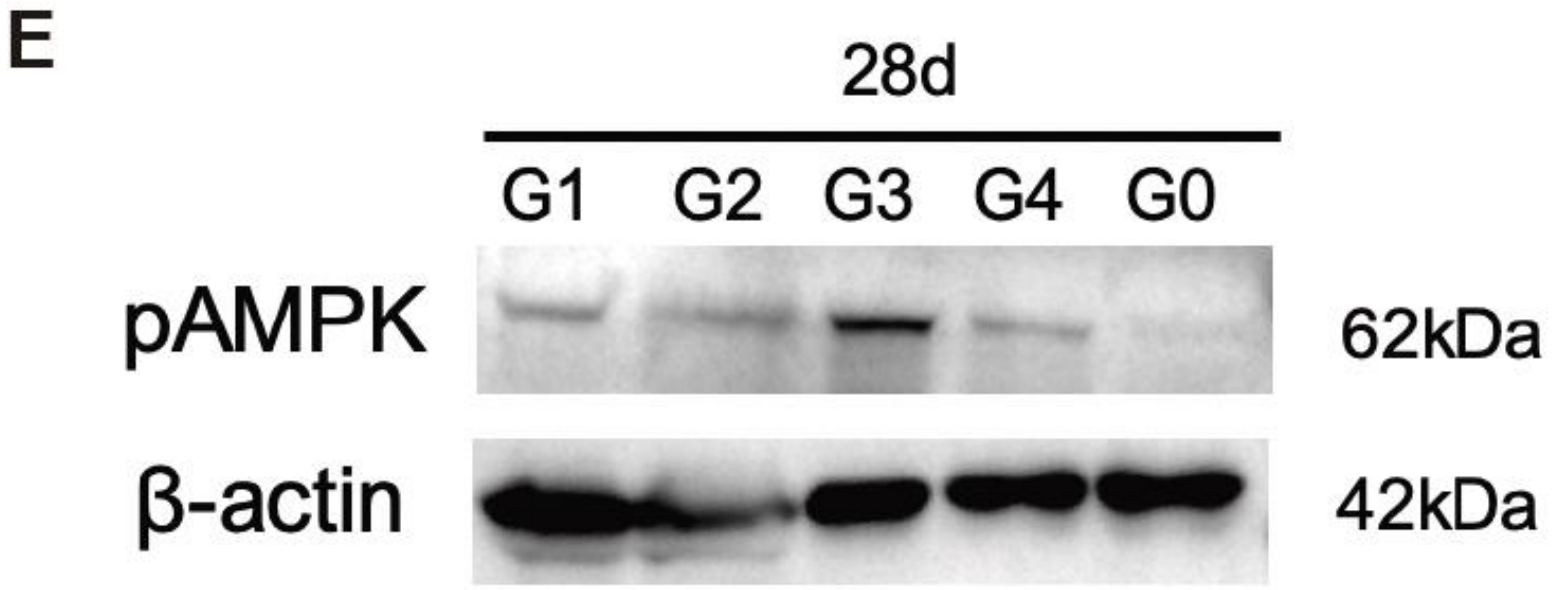

Figure 4A

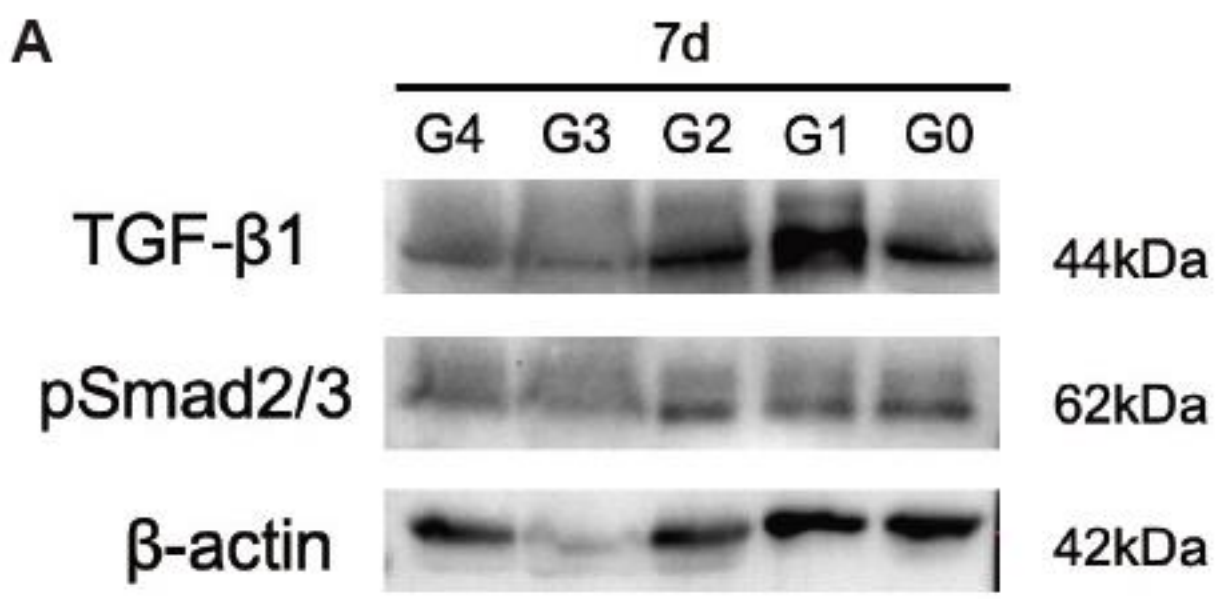

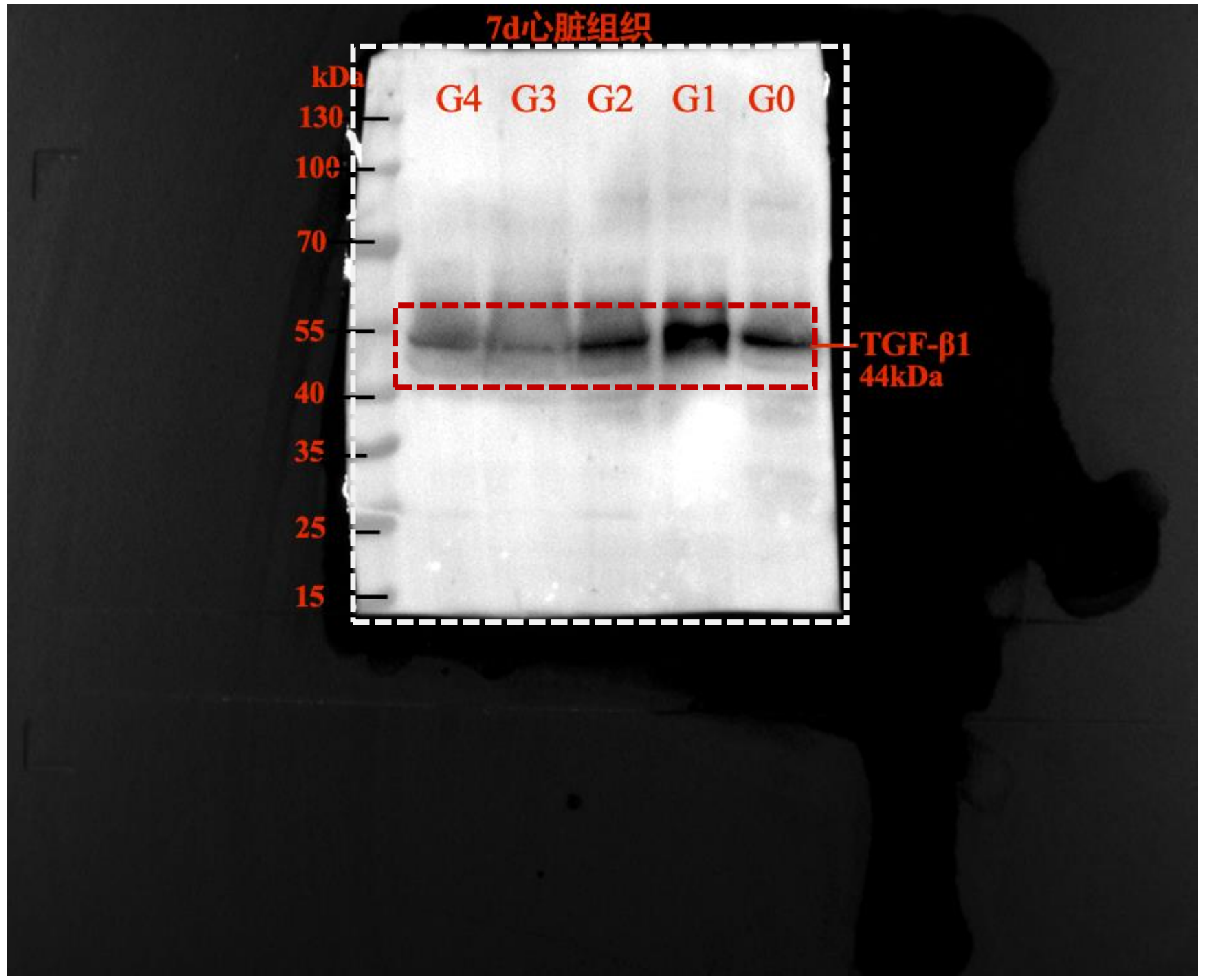

TGF-β1

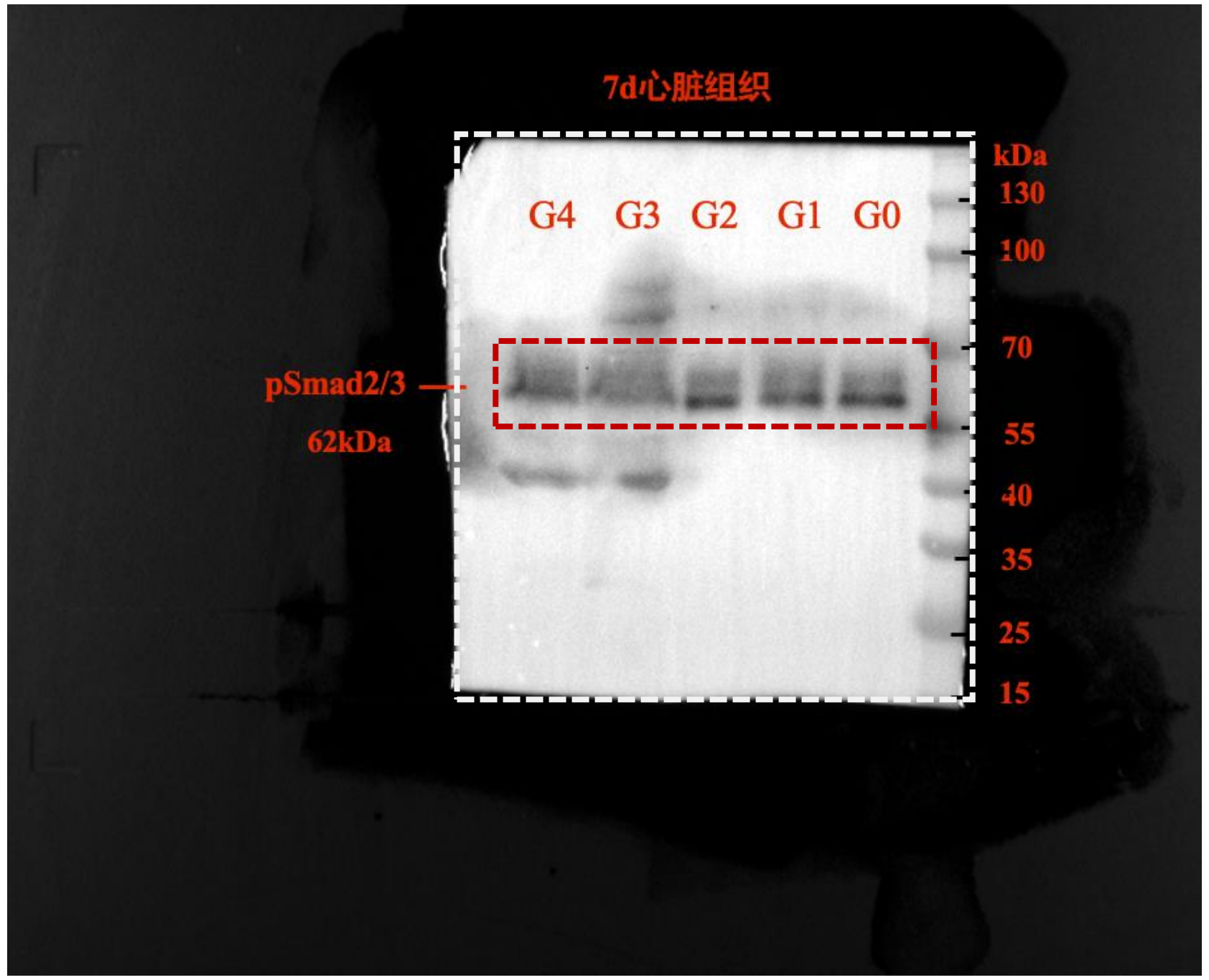

pSmad2/3

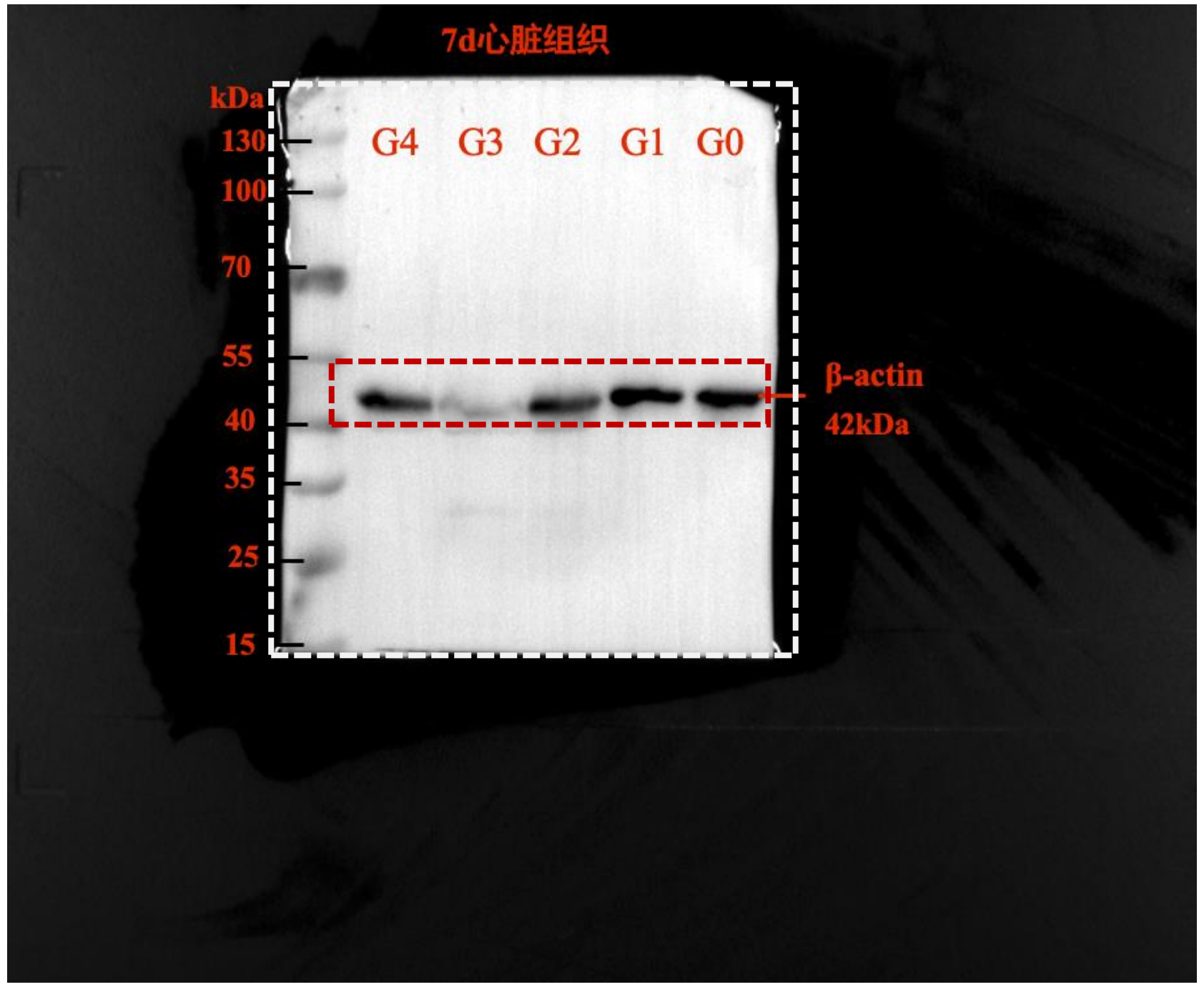

β-actin

Heart tissue collected 7 days post-MI (D7)

Figure 4B

Heart tissue collected 28 days post-MI (D28)

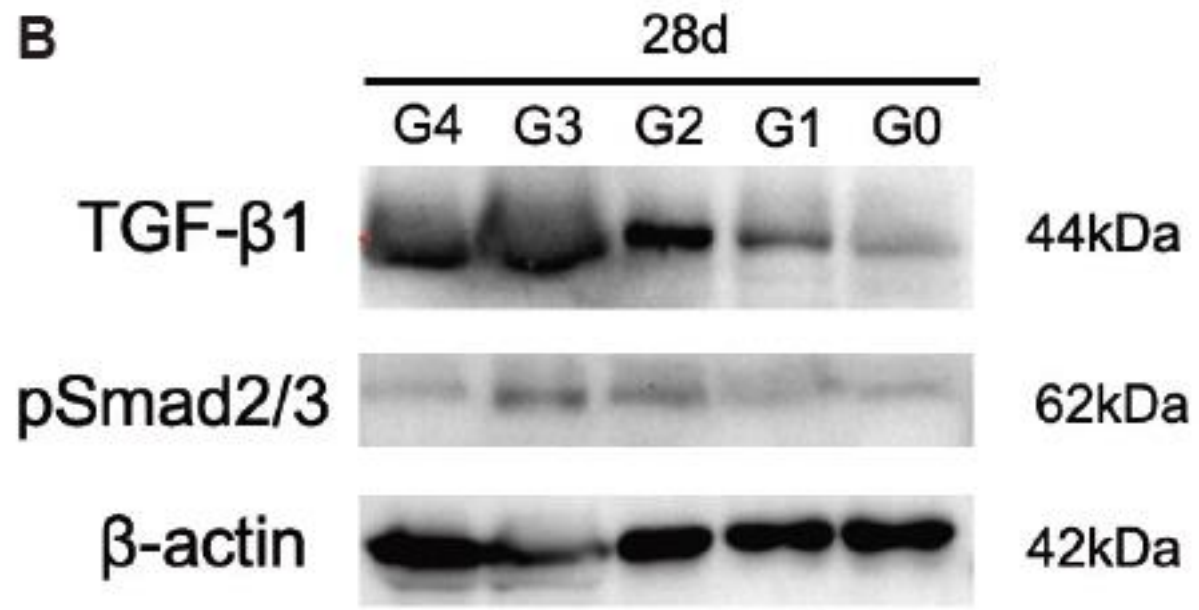

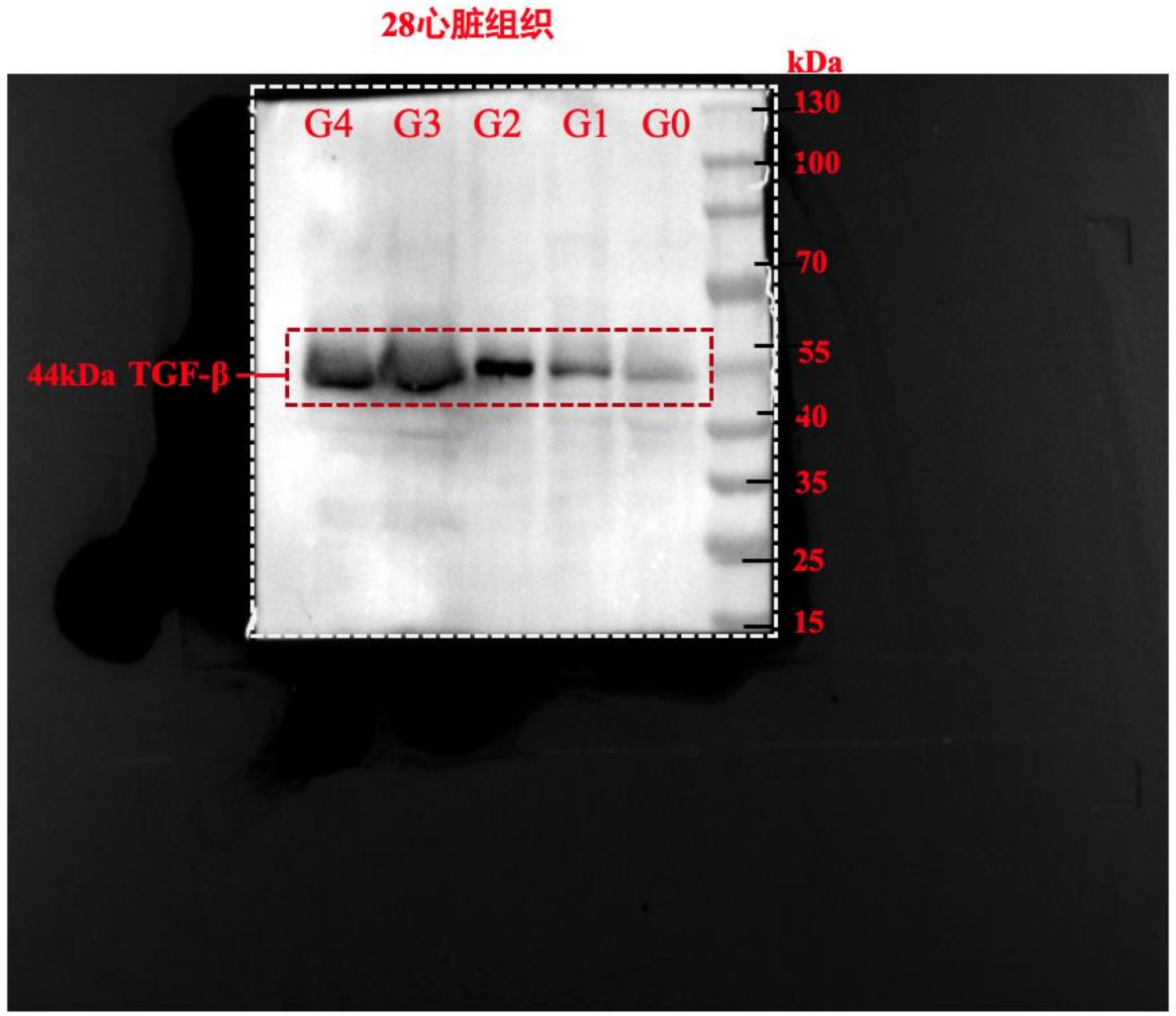

TGF-β1

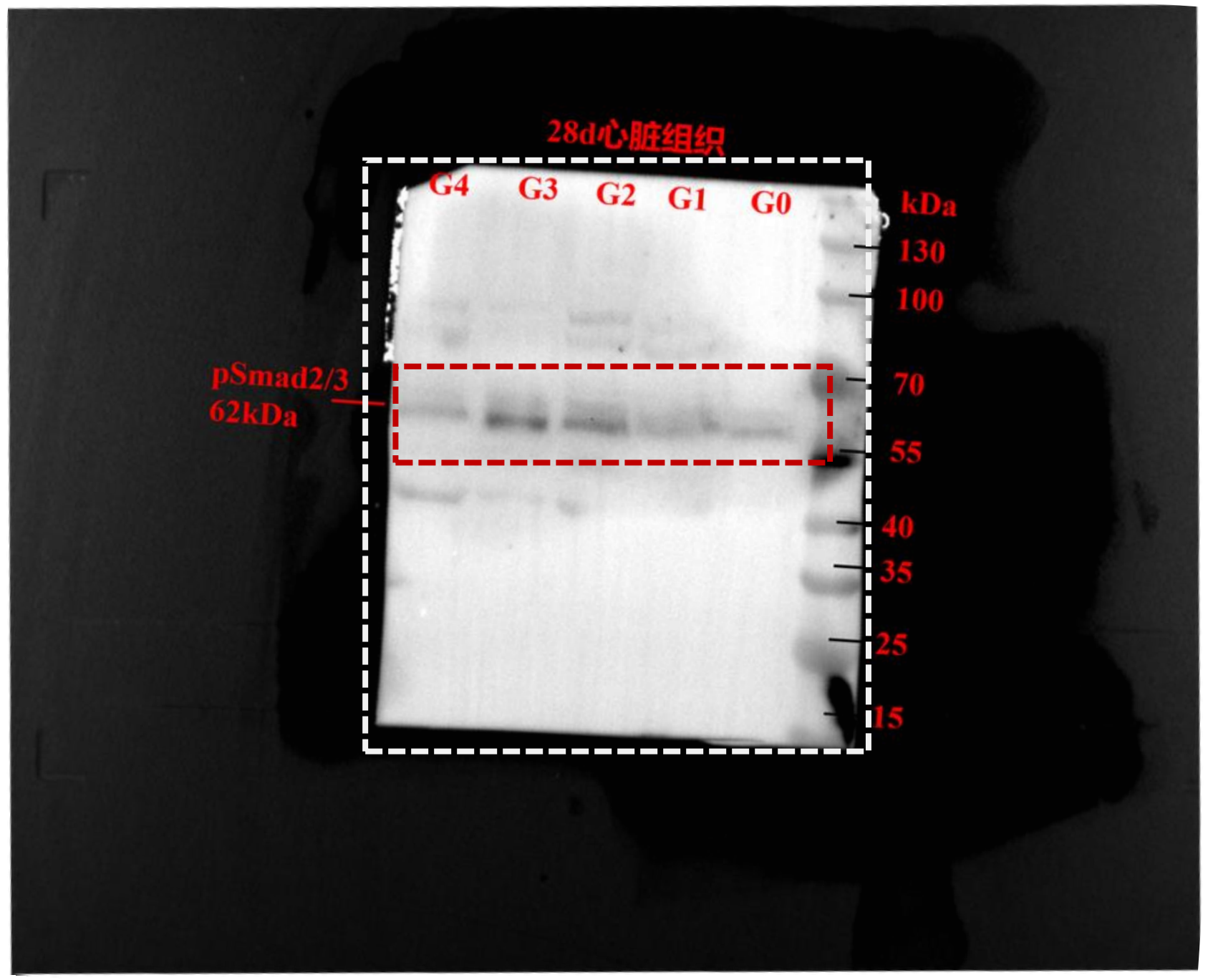

pSmad2/3

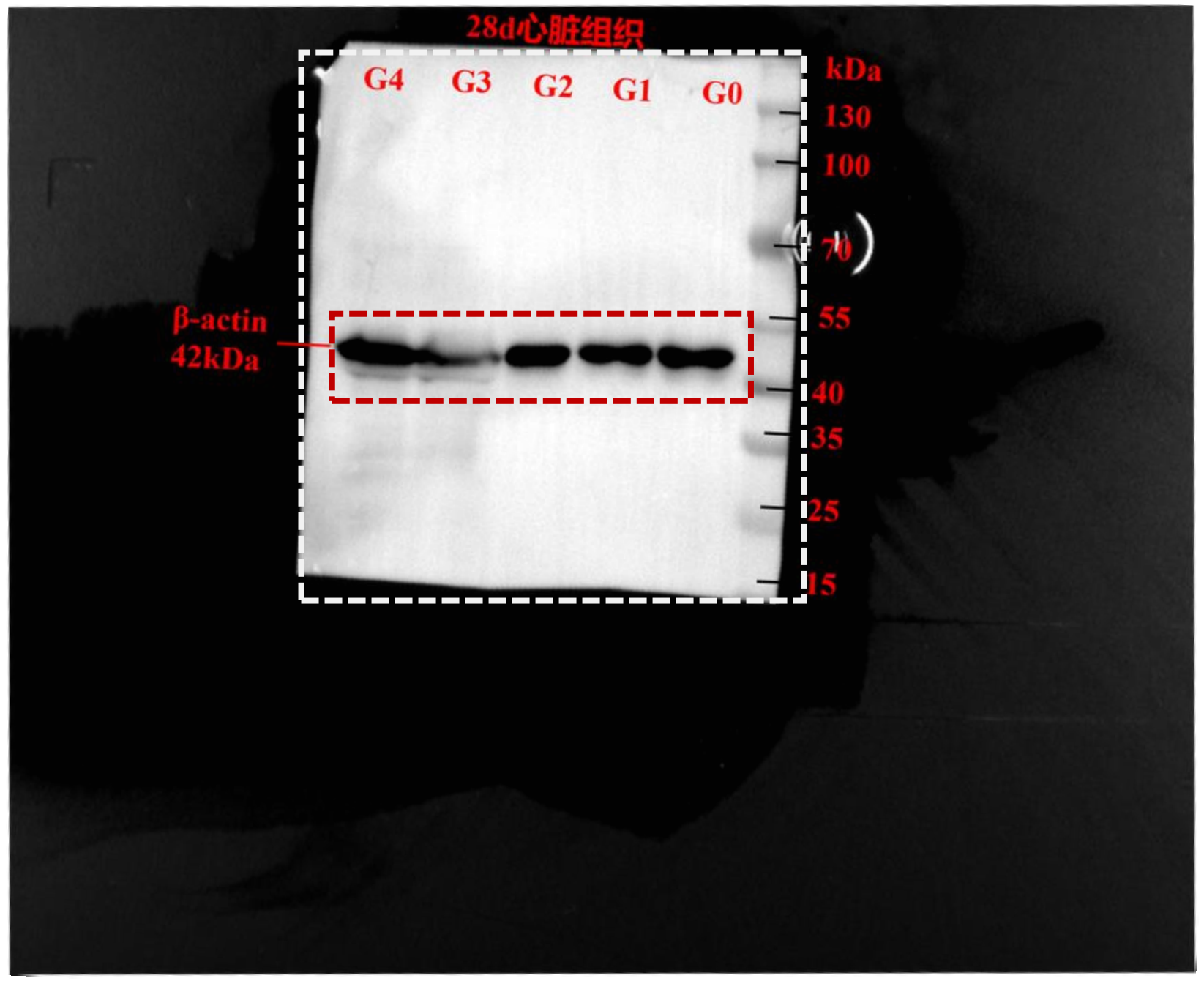

β-actin
