# Supplementary material for: Mixed short-chain fatty acids and high-fiber diet reshape SCFA profiles and attenuate post-infarction cardiac fibrosis in mice
Source: Front Cardiovasc Med. 2026 Jul 23;13:1858770. doi: 10.3389/fcvm.2026.1858770 (PMC13441702; doi:10.3389/fcvm.2026.1858770)
Supplement: Supplementary file 2 [file Table1.docx]

**Supplementary Table S1. Antibodies used for immunohistochemistry and supplementary exploratory immunoblotting.**

| REAGENT | SOURCE | IDENTIFIER | DILUTION |
| --- | --- | --- | --- |
| αSMA | Affinity | AF1032 | 1:500 |
| Collagen I | MCE | HY-P8122 | 1:500 |
| Collagen III | Proteintech | 22734-1-AP | 1:300 |
| pSmad2/3 | MCE | HY-P86139 | 1:2000 |
| pAMPK | Affinity | AF3423 | 1:500 |
| TGFβ1 | MCE | HY-P80521 | 1:500 |
