# Supplementary material for: Mixed short-chain fatty acids and high-fiber diet reshape SCFA profiles and attenuate post-infarction cardiac fibrosis in mice
Source: Front Cardiovasc Med. 2026 Jul 23;13:1858770. doi: 10.3389/fcvm.2026.1858770 (PMC13441702; doi:10.3389/fcvm.2026.1858770)
Supplement: Supplementary file 3 [file Table2.docx]

**Supplementary Table S2. Primer sequences used for quantitative real-time PCR.**

| **Tissue** | **Genes** | **Primer sequences (5’-3’)** |
| --- | --- | --- |
| **Heart** | TGF-β1 | Forward：ACCGCAACAACGCCATCTATGAG |
|  |  | Reverse：GGCACTGCTTCCCGAATGTCTG |
|  | Smad3 | Forward：AGACGCCAGTTCTACCTCCAGTG |
|  |  | Reverse：GCCAGCAGGGAAGTTAGTGTTCTC |
|  | Smad2 | Forward：GTCGTCCATCTTGCCATTCACTCC |
|  |  | Reverse：GCTCTCCACCACCTGCTCCTC |
|  | mouse IL-6 | Forward：GGGACTGATGCTGGTGACAA |
|  |  | Reverse：ACAGGTCTGTTGGGAGTGGT |
|  | Col1a1 | Forward：CCCAGTGGCGGTTATGACTT |
|  |  | Reverse：CTCAAGGTCACGGTCACGAA |
|  | Col3a1 | Forward：ACGAGGTGACAAAGGTGAAACTGG |
|  |  | Reverse：AGAACCTGGAGGACCTGGATTGC |
|  | AMPK | Forward：CTTTGGCAGTTGCCTACCAT |
|  |  | Reverse：GGCTTGTCGCCAAATAGAAA |
| **Intestine** | ZO1 | Forward：TCCCACAAGGAGCCATTCCTG |
|  |  | Reverse：GGGCTCAGCAGAGTTTCACCT |
|  | Claudin | Forward：TAAGGCACGGGTAGCACTCAC |
|  |  | Reverse：GATGTTGGCGAACCAGCAGAG |
|  | Occludin | Forward：AGGAGGACTGGGTCAGGGAATA |
|  |  | Reverse：TGACGTCGTCTAGTTCTGCCTG |
